# Supplementary material for: The Evolution and Characterization of the RNA Interference Pathways in Lophotrochozoa
Source: Genome Biol Evol. 2024 May 7;16(5):evae098. doi: 10.1093/gbe/evae098 (PMC11114477; doi:10.1093/gbe/evae098)
Supplement: evae098_Supplementary_Data [file evae098_supplementary_data.zip › Supplementary_materials.zip!/selected_miRNAs/NoGe_selected_miRNAs/known_miRNA_families_pdf_files/NMRB01000765.1_Notospermus_geniculatus_strain_Ushimado_scaffold765,_whole_genome_shotgun_sequence_20624.pdf]

Provisional ID	:	NMRB01000765.1_Notospermus_geniculatus_strain_Ushimado_scaffold765,_whole_genome_shotgun_sequence_20624
Score total	:	4960496.9
Score for star read(s)	:	3.9
Score for read counts	:	4960488.5
Score for mfe	:	1.5
Score for randfold	:	0
Score for cons. seed	:	3
Total read count	:	9729796
Mature read count	:	9611509
Loop read count	:	0
Star read count	:	118287

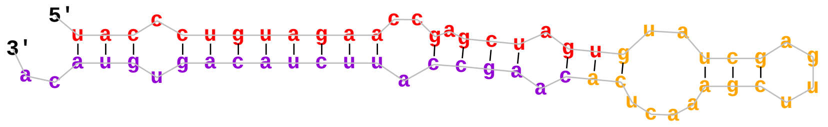

Genome coordinates: NMRB01000765.1 112443-112505

pre-miRNA sequence:
UACCCUGUAGAACCGAGCUAGUUACCCUGUAGAACCGAGCUAGUGUAUCGAGUUCGAAACUCACAAGCCAUUCUACAGUGUACA

Mature sequence: UACCCUGUAGAACCGAGCUAGU

Aligned mature sequence of the *N. geniculatus* pre-miRNA with the top 5 hits from the BLAST search against the pre-miRNA mirGeneDB set:

```
                                *****************  **
    Nge_Mir-10-P7              -uacccuguagaaccgagcuagu-
    Cgi-Mir-10-P5_5p           uuacccuguagaaccgagcgagug
    Efe-Mir-10-P1j_5p          -uacccuguagaaccgagcuugu-
    Cte-Mir-10-P7_5p           -gacccuguagaaccgagcuugug
```
